# Repurposing of Clomiphene Citrate and Its Antileishmanial and Antifungal Activities: *In Silico* and *In Vitro* Studies

**DOI:** 10.3390/biom16081133

**Published:** 2026-08-03

**Authors:** Leandro Josuel da Costa Santos, Érika de Araújo Abi-chacra, Rita de Cássia Vianna de Carvalho, Lucas Malaquias França, Denise Andrade do Nascimento, Fernando Aécio de Amorim Carvalho, Gabriel Zazeri, André Luis Menezes Carvalho

**Affiliations:** 1 Postgraduate Program in Pharmaceutical Sciences, Federal University of Piauí, Campus Ministro Petrônio Portela, Teresina 64049-550, PI, Brazil; leandrosantos.educ@gmail.com (L.J.d.C.S.); rita.carvalho@undb.edu.br (R.d.C.V.d.C.); famorim@ufpi.edu.br (F.A.d.A.C.); 2 Department of Parasitology and Microbiology, Federal University of Piauí, Campus Ministro Petrônio Portela, Teresina 64049-550, PI, Brazil; abichacra@ufpi.edu.br; 3 University Center Higher Education Unit Dom Bosco, Curitiba 81010-000, PR, Brazil; 4 Undergraduate Course in Pharmacy, Federal University of Piauí, Campus Ministro Petrônio Portela, Teresina 64049-550, PI, Brazil; lucasmalaquiasfranca@gmail.com; 5 Department of Physics, Federal University of Roraima (UFRR), Boa Vista 69310-000, RR, Brazil; denise.nascimento@ufrr.br

**Keywords:** drug repurposing, clomiphene, ADME, cutaneous leishmaniasis, *Candida albicans*, *in silico* study

## Abstract

This study investigated the repurposing potential of clomiphene citrate as a topical therapeutic candidate for tegumentary leishmaniasis (TL) and candidiasis. Comparative *in silico* analyses were performed to evaluate absorption, distribution, metabolism, excretion, and toxicity (ADMET) properties in comparison with meglumine antimoniate and amphotericin B. In addition, molecular docking was conducted to investigate the interaction of clomiphene with *Leishmania* spp. arginase and the Sap5 protease of *Candida albicans*. Clomiphene exhibited a favorable physicochemical profile, characterized by high lipophilicity, moderate skin permeability, and predicted oral bioavailability and intestinal absorption superior to those of the reference drugs. Toxicological predictions also indicated a lower systemic toxicity profile compared with conventional therapies. Molecular docking revealed favorable binding energies and interactions with key residues within the active sites of both targets. Experimental assays demonstrated that clomiphene inhibited the early stages of *C. albicans* biofilm formation and exhibited potent leishmanicidal activity against *L. amazonensis* promastigotes, with a high selectivity index in macrophages. Together, these findings indicate that clomiphene citrate represents a promising candidate for further investigation as a potential therapeutic agent against tegumentary leishmaniasis and candidiasis.

## 1. Introduction

Leishmaniasis is a group of infectious diseases caused by protozoan parasites of the genus *Leishmania*, transmitted by phlebotomine sandflies, with clinical forms that affect the skin, mucous membranes, and internal organs. Among these is Tegumentary Leishmaniasis (TL), which is characterized by chronic, ulcerated skin lesions that lead to deformities and psychosocial impacts [1].

Globally, the cutaneous and mucosal forms are endemic in most countries, with hundreds of thousands of new annual cases, making them highly relevant neglected tropical diseases [2]. In Brazil, in 2024, approximately 311,968 cases of TL were reported, with an average detection rate exceeding 6 cases per 100,000 inhabitants. The highest concentration was found in the North and Northeast regions [3,4].

The epidemiological profile indicates a higher prevalence among adults, particularly men involved in rural or extractive activities [5]. The presence of these diseases in vulnerable populations underscores the need for integrated surveillance and control actions [6]. Pentavalent antimonials remain the first-line therapy; however, severe adverse effects have been reported, including cardiotoxicity, acute pancreatitis, pancytopenia, and shock [7]. Alternatives include meglumine antimoniate, which is effective but presents considerable toxicity [8]. Amphotericin B (AmB), in either conventional or liposomal formulations, is recommended for severe or refractory cases because of its high efficacy, although its clinical use is limited by nephrotoxicity [4,9]. Therefore, treatment selection must consider the *Leishmania* species, clinical presentation, and patient-specific conditions, following current therapeutic guidelines [10].

In patients with TL, particularly those presenting comorbidities or immunosuppression, clinical management may be further complicated by opportunistic infections [11,12], including those caused by fungi of the genus *Candida*. These therapeutic limitations reinforce the need to identify safer and more effective treatment alternatives [4].

In this context, drug repurposing has emerged as an attractive strategy for accelerating the discovery of new therapeutic applications while reducing development time and costs. Selective estrogen receptor modulators (SERMs), in particular, constitute a promising class of compounds because they have demonstrated antibacterial, antifungal, antiviral, and antiparasitic activities in addition to their well-established endocrine effects [13]. Reflecting this broad spectrum of biological activities, SERMs such as tamoxifen and raloxifene have already demonstrated promising antileishmanial activity [14].

Clomiphene, another member of the SERM family traditionally used for ovulation induction, represents a potential candidate for therapeutic repurposing in cutaneous leishmaniasis. Its pronounced lipophilicity favors interaction with biological membranes and may facilitate topical delivery and intracellular access to parasites and fungal cells [15].

The investigation of clomiphene through computational approaches should focus on molecular targets of recognized clinical importance. In *Leishmania*, L-arginase is an essential enzyme involved in parasite survival and replication [16,17], whereas, in *Candida albicans*, secreted aspartyl proteinase 5 (Sap5) plays a central role in adhesion, tissue invasion, and biofilm formation [18,19]. Both proteins represent validated therapeutic targets for the discovery of novel antiparasitic and antifungal compounds.

Despite the growing interest in drug repurposing for neglected infectious diseases, there remains limited evidence integrating comprehensive ADMET prediction, molecular docking against both *Leishmania* arginase and *Candida albicans* Sap5, and experimental validation through antileishmanial and antifungal assays for clomiphene. Most previous studies have focused either on isolated computational analyses or on individual biological evaluations, leaving a gap in the integrated assessment of clomiphene as a multitarget therapeutic candidate.

In this scenario, computational approaches represent powerful tools for identifying compounds with dual antileishmanial and antifungal potential. Molecular docking and *in silico* screening enable the evaluation of structural complementarity and physicochemical compatibility between small molecules and biological targets, supporting the selection of candidates with greater therapeutic potential [20,21].

Therefore, the novelty of the present study lies in integrating complementary *in silico* and *in vitro* approaches to comprehensively evaluate the therapeutic repurposing potential of clomiphene. Specifically, we combined ADMET prediction, molecular docking against two clinically relevant targets (*Leishmania* arginase and *Candida albicans* Sap5), ATR-FTIR characterization, antileishmanial and antifungal biological assays, cytotoxicity evaluation, and selectivity analysis. This integrated strategy provides a more comprehensive assessment of clomiphene as a promising candidate for the treatment of neglected infectious diseases than studies based solely on computational predictions or experimental assays.

Thus, the present study aims to simultaneously evaluate the interaction of clomiphene with biologically relevant molecular targets while characterizing its pharmacokinetic, toxicological, and biological profiles, thereby providing a comprehensive rationale for its therapeutic repurposing as a potential antiparasitic and antifungal agent.

## 2. Materials and Methods

### 2.1. Data Source and Computational Tools

**Figure 1:**
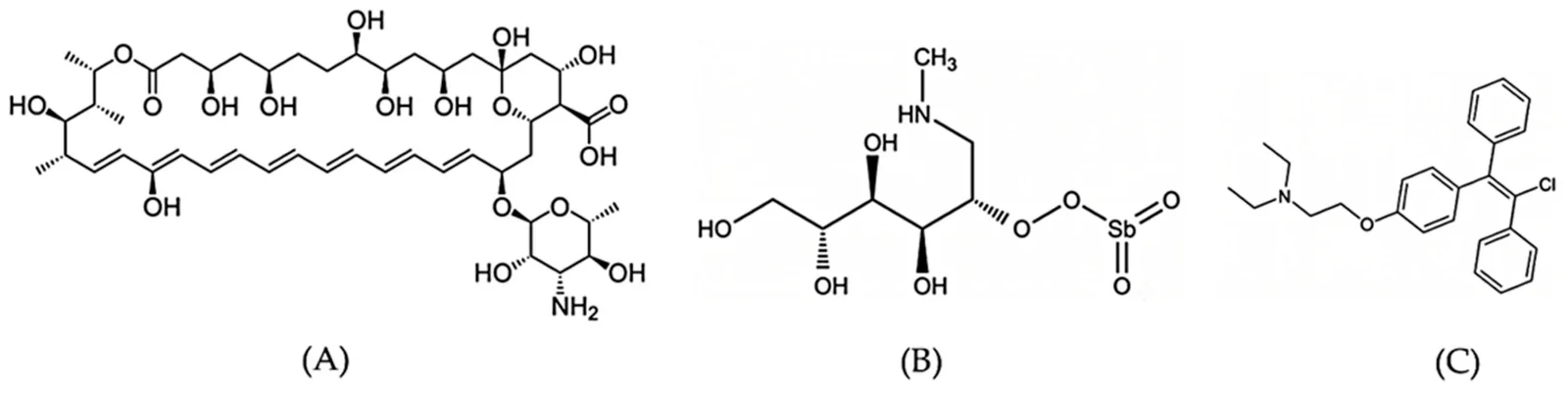
Chemical structures of the molecules employed in the *in silico* study: (**A**) amphotericin B, (**B**) meglumine antimoniate, and (**C**) clomiphene.

The PubChem database (https://pubchem.ncbi.nlm.nih.gov/, accessed on 10 May 2026) is a platform that explores integrated data on chemical compounds, substances, and bioassays, providing information on molecular interactions and biological targets [22]. From this database, the molecular structures of amphotericin B, meglumine antimoniate, and clomiphene were obtained (Figure 1), along with their respective SMILES strings, to be used for comparison in the *in silico* study.

The atomic coordinates of the protein–ligand complexes were obtained from the RCSB Protein Data Bank (RCSB PDB; https://www.rcsb.org/, accessed on 11 May 2026), a database that stores three-dimensional structures of biological macromolecules (proteins, nucleic acids, and molecular complexes). From these coordinates, the corresponding three-dimensional structures were visualized and analyzed using PyMOL software in the Edu-PyMOL 3.1 version. [23]. For the *in silico* evaluation of the compounds, ADME prediction platforms were utilized; these platforms forecast the behavior of a molecule within a living organism [24] and were used in this study to predict the behavior of the amphotericin B, meglumine antimoniate, and clomiphene molecules.

The SwissADME platform (https://www.swissadme.ch/, accessed on 11 May 2026), developed by the Swiss Institute of Bioinformatics (SIB), was used to predict the physicochemical, pharmacokinetic, and medicinal chemistry properties of small molecules. Additionally, the platform features the BOILED-Egg model, a chemometric method that provides a rapid and visual prediction of the probability that a small molecule will exhibit good gastrointestinal absorption and/or the ability to cross the blood–brain barrier, based solely on its lipophilicity (LogP) and polarity (PSA) [25].

The ProTox-3 platform (https://tox-new.charite.de/protox_II, accessed on 13 May 2026) was employed to predict acute oral toxicity (LD_50_), toxicity classes, and the potential for hepatotoxicity, mutagenicity, cytotoxicity, carcinogenicity, and immunotoxicity [26,27,28,29].

The PreADMET tool (https://preadmet.webservice.bmdrc.org/, accessed on 16 May 2026) was used to complement the pharmacokinetic and toxicity data predictions. It predicted plasma protein binding, permeability in Caco-2 cells and MDCK cells (utilized as biological barrier models), mutagenic toxicity, hepatic metabolism, and interactions with cytochrome P450 (CYP450) and other CYP isoforms. Additionally, ecotoxicity predictions were performed for algae, *daphnia*, *medaka*, and *minnow* [30].

The PASS Online tool (http://www.way2drug.com/passonline, accessed on 16 May 2026) was utilized to provide the probabilities of biological activity (Pa) and inactivity (Pi) across a wide range of pharmacological effects. It was employed to predict activities related to analgesia, anti-inflammatory action, COX-2 inhibition, and the modulation of TRPV1, GABA, and sodium channels [31].

To further complement the study, the GUSAR platform (https://way2drug.com/Gusar/, accessed on 16 May 2026) was also utilized. This tool is capable of predicting LD_50_ values (mg/kg) across different administration routes (oral, intravenous, intraperitoneal, and subcutaneous), providing toxicological categorization according to OECD standards [32]. It was employed to estimate acute oral toxicity using SMILES-based QSAR modeling.

Furthermore, the admetSAR 3.0 tool (https://lmmd.ecust.edu.cn/admetsar3/, accessed on 17 May 2026) was also employed, utilizing machine learning-based computational models to estimate parameters related to the absorption, distribution, metabolism, excretion, and toxicity (ADME) profile, thereby enabling the integrated prediction of pharmacokinetic properties for the analysed compounds [33].

### 2.2. Molecular Docking Studies

**Figure 2:**
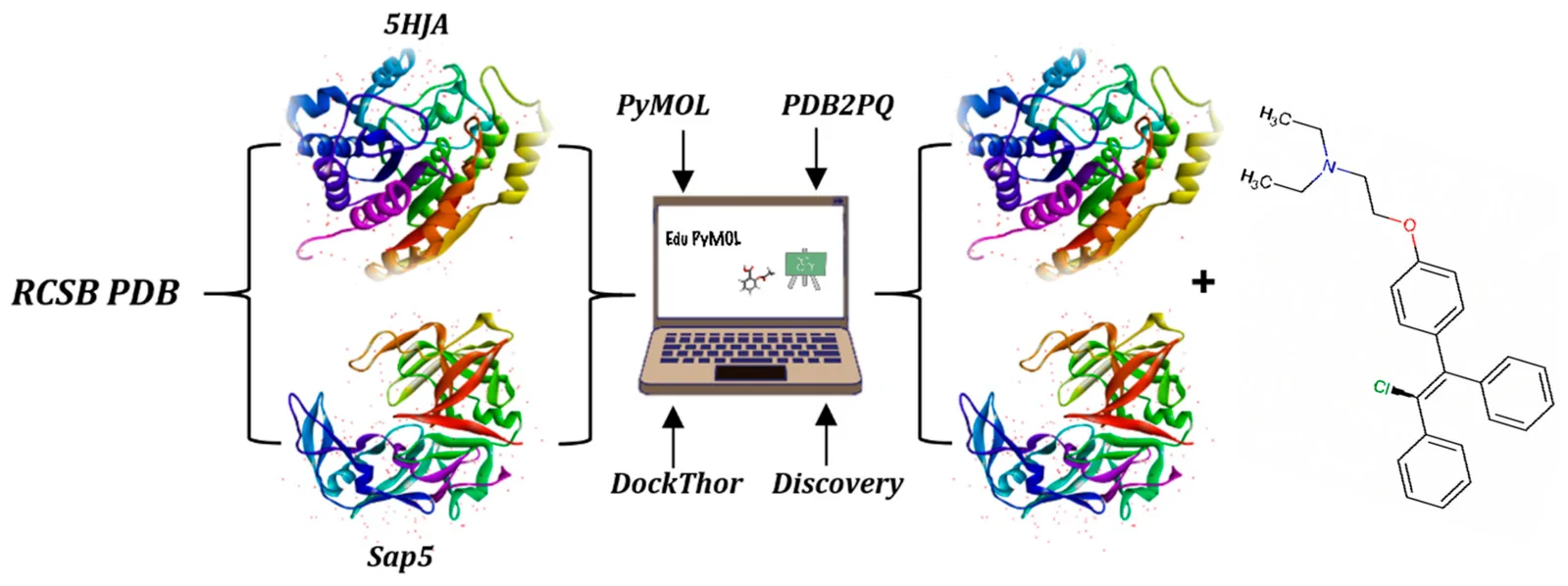
Methodological workflow of the molecular docking of clomiphene into arginase (5HJA) and (Sap5).

The molecular docking study was conducted using L-arginase and Sap5 as receptor proteins. Their three-dimensional structures were obtained from the Protein Data Bank (PDB) and used to evaluate the interactions between the target proteins and the tested ligands (Figure 2). 

The protein structures were retrieved from the Protein Data Bank (PDB) and prepared prior to the molecular docking simulations. Initially, the co-crystallized ligands were removed from the receptor structures using PyMOL to allow docking of the ligands. The software was also used to remove non-essential crystallographic water molecules, inspect the protein structures, and define the coordinates of the native binding sites based on the position of the co-crystallized ligands. To validate the docking protocol, the co-crystallized ligands were redocked into their respective binding sites. The root mean square deviation (RMSD) values between the crystallographic and predicted co-crystallized poses were 1.4145 Å for Sap5 (PDB ID: 2QZX) and 1.6166 Å for L-Arginase (PDB ID: 5HJA), both below the commonly accepted threshold of 2.0 Å, confirming the reliability of the docking protocol (Figure S1). After the validation of the docking protocols, the native binding sites were then used to define the docking search space. For Sap5 (PDB ID: 2QZX), the grid box was centered at x = 17, y = 35, z = 31, with dimensions of 20 × 20 × 20 Å. For L-Arginase (PDB ID: 5HJA), the grid box was centered at x = −21, y = −7, z = 34, with dimensions of 21 × 21 × 21 Å. In both cases, a grid spacing of 0.25 Å was employed (Figure S2). Molecular docking calculations were performed using the DockThor platform with the standard genetic algorithm parameters, including 1,000,000 energy evaluations, a population size of 750, and 24 independent docking runs for each ligand–protein complex. Protein structures were treated as rigid, whereas ligands were considered fully flexible during the simulations. The final docking pose was selected based on the lowest predicted binding free energy (ΔG) provided by the DockThor scoring function. The resulting protein–ligand complexes were analyzed using Discovery Studio Visualizer (v.21). This software was employed to visualize the docking poses and characterize the intermolecular interactions, including hydrogen bonds, hydrophobic contacts, π–π interactions, and π–cation interactions. The binding poses were selected based on their docking energy scores, and the interaction profiles obtained were used for subsequent structural and energetic analyses.

### 2.3. FTIR

Infrared spectroscopy was used to identify the characteristic bands of the drug. The spectral analysis of the sample obtained by attenuated total reflectance-Fourier transform infrared spectroscopy (ATR-FTIR) was performed using an Agilent Cary 630 FTIR spectrometer (Agilent Technologies Inc., 5301 Stevens Creek Blvd, Santa Clara, CA, USA) equipped with a diamond crystal, single reflection, top plate, and pressure arm, utilizing Agilent MicroLab software 5.8.

The measurement mode was transmittance within the mid-infrared region (4000–500 cm^−1^). The number of scans was 64, with a resolution of 2 cm^−1^.

### 2.4. Evaluation of Clomiphene Citrate Activity Against C. albicans Biofilm Synthesis

**Figure 3:**
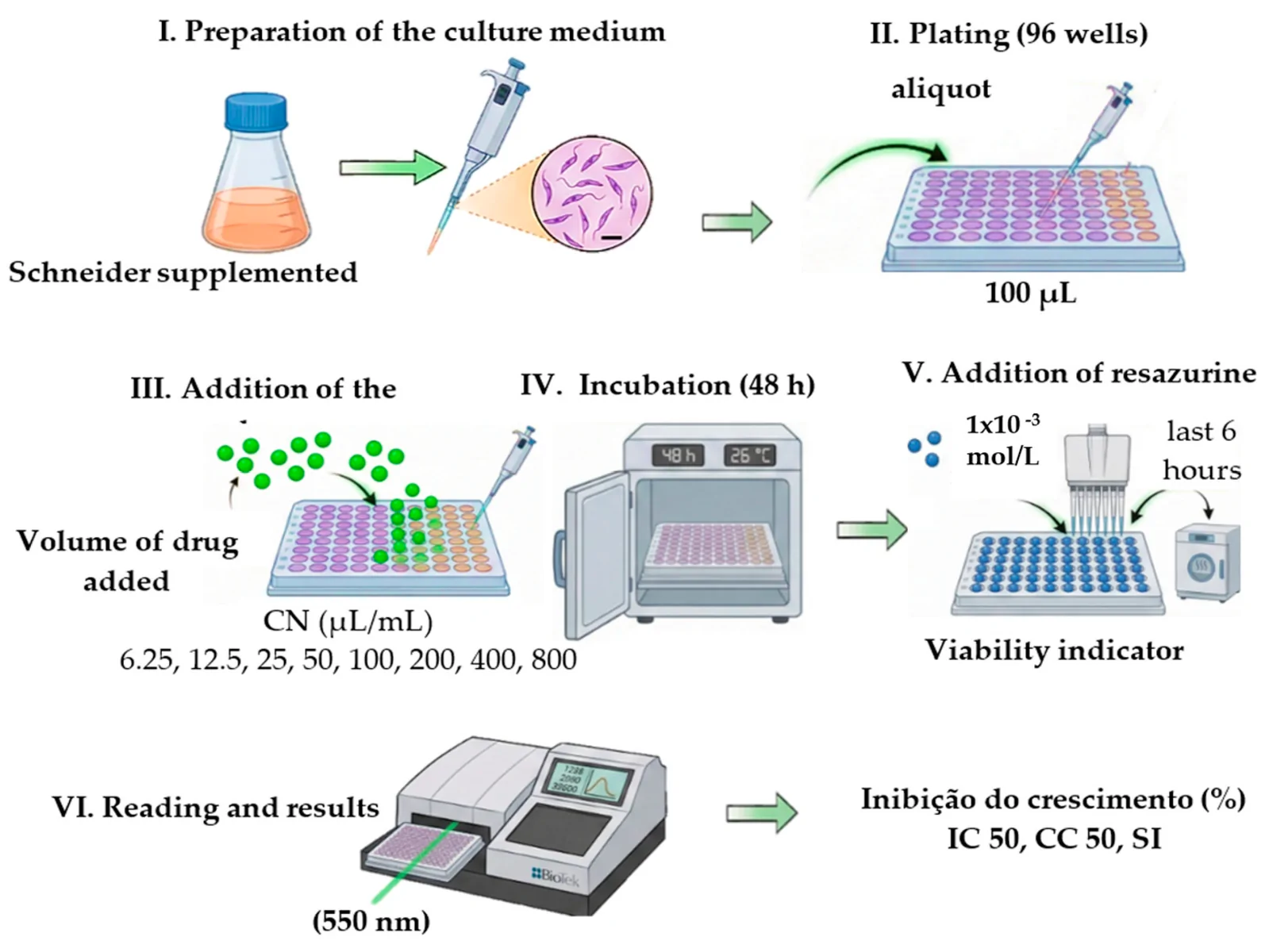
Methodological workflow of experimental design and laboratory stages for the quantification of *C. albicans* biofilm biomass. INH: inhibition of biofilm formation; ABS_t_: absorbance of the test at 590 nm; ABS_c_: absorbance of the control at 590 nm.

The experimental design followed three distinct stages: pre-treatment, treatment, and post-treatment (Figure 3), aiming to understand the formulation’s dynamics of action as previously investigated in other studies [34,35,36]. The pre-treatment stage aimed to evaluate the influence of the active compounds on the initial adhesion capacity and virulence of the yeasts prior to seeding in 96-well plates.

To evaluate the effect of clomiphene citrate on the inhibition of biofilm formation, yeast fungi of the species *Candida albicans* (*C. albicans*) (ATCC-10231) were used. In the pre-treatment stage, fungi (1 × 10^6^ cells) pre-treated with the drug (10, 25, 50, and 100 µg/mL) for 24 h were transferred to a 96-well polystyrene microtiter plate and incubated for 48 h at 37 °C to allow the formation of a mature biofilm. For the co-treatment stage, fungi (1 × 10^6^ cells) were incubated with the drug (10, 25, 50, and 100 µg/mL) during the 48-h biofilm formation period in the polystyrene plate. Finally, in the post-treatment stage, fungi (1 × 10^6^ cells) were transferred to the polystyrene plate and incubated for 48 h to form a mature biofilm, after which the system was treated with the drug (10, 25, 50, and 100 µg/mL) for an additional 24 h. Subsequently, the wells were washed three times with saline solution to remove non-adherent cells. The resulting biofilm biomass was then evaluated using the crystal violet assay [37]. The wells were incubated with crystal violet stain at room temperature for 5 min. Then, the systems were washed three times with distilled water to remove excess stain, followed by the addition of ethanol for 5 min. The de-staining solution was transferred to 96-well plates, and the absorbance was measured at 550 nm using an ELISA microplate reader (EZ Read 400, Biochrom Ltd., Cambridge, UK). 

The quantification of fixed cells stained on the well walls was performed using a microplate spectrophotometer (BioTek–EL800) at 590 nm, acquired by BioTek Instruments, Inc., Winooski, VT, USA. In parallel, a negative control condition was established consisting solely of the culture medium and the fungal suspension. Each assay was performed in triplicate, and the final data were represented by the mean of the read absorbances and presented graphically. The percentage of biofilm suppression was determined by Equation (1) according to the methodology described in previous studies [38,39].

(1) $$\%\;INB=100-\left(\;\frac{ABS\;t}{ABS\;c}\;\right)\times 100$$

### 2.5. Investigation of Clomiphene Citrate Activity Against L. amazonensis Promastigotes, Cytotoxicity, and Selectivity Index (SI) in Macrophages

**Figure 4:**
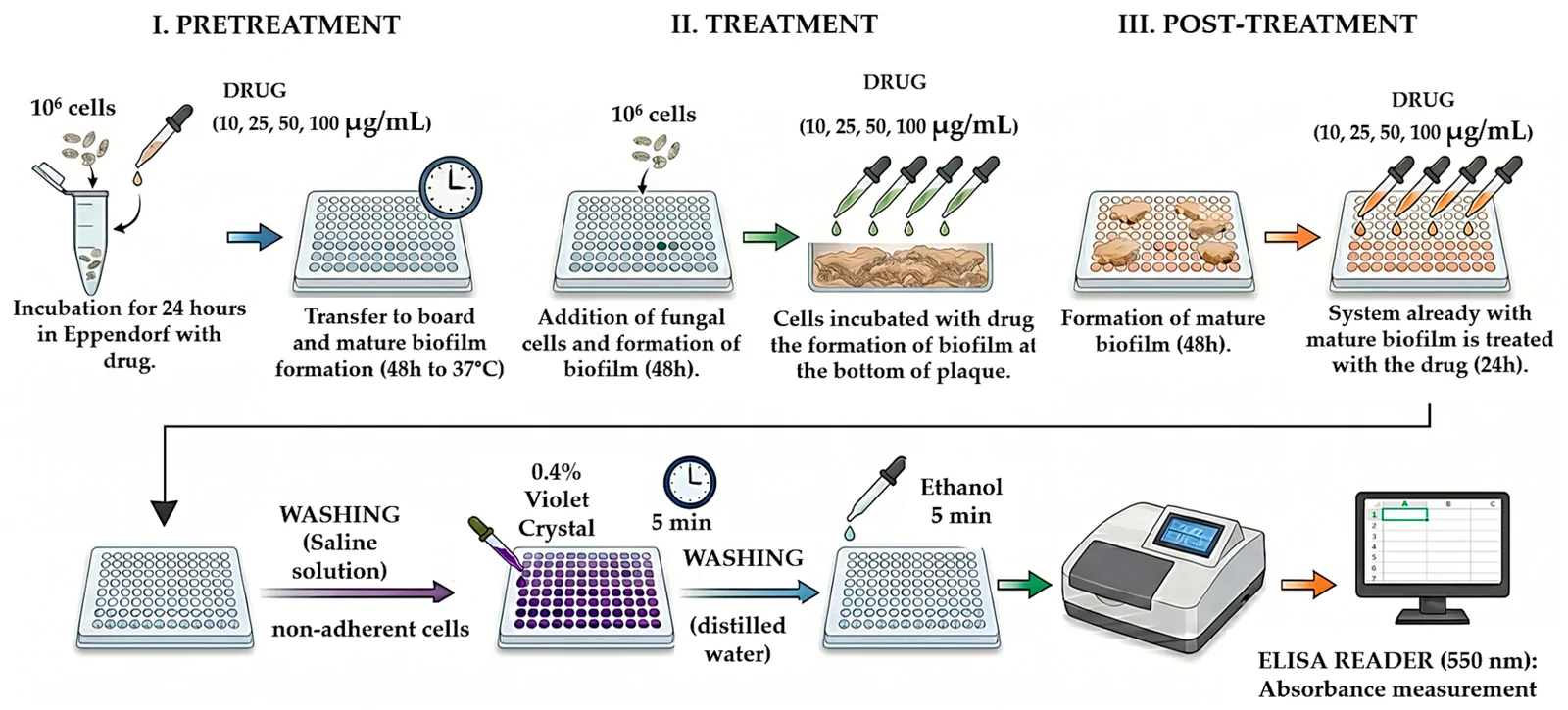
*In vitro* methodological procedure of the antileishmanial activity of clomiphene citrate against *L. amazonensis* promastigotes.

The antileishmanial potential of clomiphene was tested against *L. amazonensis* specimens in the late exponential growth phase, as shown in the flowchart (Figure 4). The protozoa, maintained in enriched Schneider’s medium, were subjected to a drug dosage gradient at the concentrations designated by the protocol of 6.25; 12.5; 25; 50; 100; 200; 400; and 800 μg/mL) in 96-well microplates, which were kept in a BOD incubator for at least 48 h at a temperature of 26 °C. Optical density was measured via spectrophotometry at 550 nm, utilizing amphotericin B (2 μg/mL) as the positive control (maximum efficacy) and Schneider’s culture medium (1 × 10^6^ cells/well) as the reference for full growth. To ensure data accuracy, the residual signal of the medium components (blank) was subtracted from all values, allowing the precise calculation of the percentage of parasite proliferation suppression relative to the untreated group.

Cell viability analysis following clomiphene treatment was conducted using the MTT colorimetric assay. Initially, a density of 2 × 10^5^ of RAW macrophages was distributed into each well of 96-well plates using 100 μL of RPMI medium (supplemented with 10% FBS, as well as 10,000 IU of penicillin and 1000 IU of streptomycin). The system was maintained in a controlled environment at 37 °C under a 5% CO_2_ atmosphere for 4 h to allow for cell adherence to the surface. Subsequently, the supernatant was removed to eliminate non-adherent cells. The compound was then introduced in serial dilutions within the culture medium, establishing an eight-level gradient (ranging from 0.78 to 100.00 μg/mL), followed by further incubation in an incubator (37 °C and 5% CO_2_) for a 48-h period. To quantify the cytotoxic effect, a 10% MTT solution (5 mg/mL) in 100 µL of RPMI was added, followed by re-incubation for an additional 4 h under the same thermal and gaseous conditions.

Following supernatant removal, 100 μL of DMSO was added under constant agitation for 30 min to solubilize the formazan crystals. Optical density was measured at 550 nm using a microplate reader. Finally, the selectivity index (SI) was calculated as the ratio of the cytotoxic concentration for 50% of cells (CC_50_) in macrophages to the half-maximal inhibitory concentration (IC_50_) against promastigotes [40,41].

## 3. Results and Discussion

### 3.1. Physicochemical and Pharmacokinetic Properties

**Figure 5:**
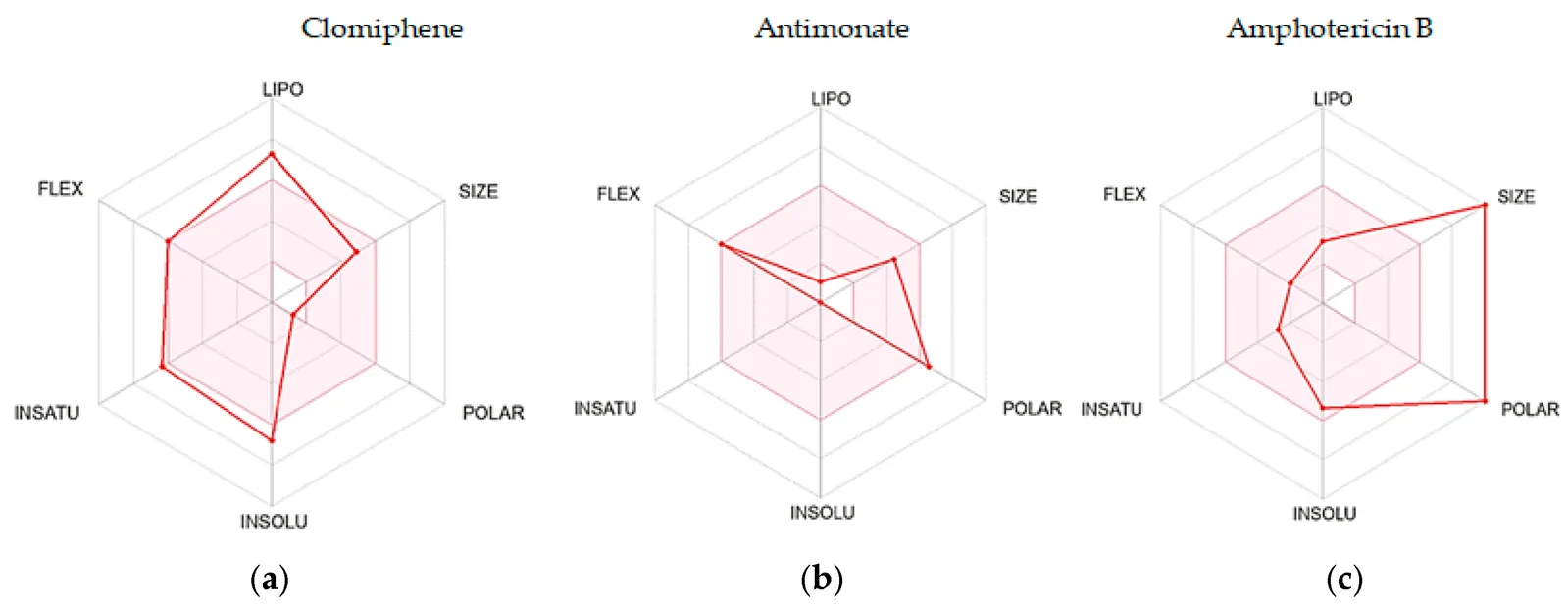
Oral bioavailability radar for the drugs clomiphene, antimoniate, and amphotericin B. (**a**): Oral bioavailability radar of clomiphene; (**b**): bioavailability radar of antimoniate; and (**c**): oral bioavailability radar of amphotericin B.

SwissADME predictions (Figure 5) indicated distinct physicochemical profiles for clomiphene, meglumine antimoniate, and amphotericin B. Clomiphene showed high lipophilicity (XLOGP3 ≈ 6.0), whereas antimoniate and amphotericin B presented markedly lower values (−2.79 and 0.39, respectively). Despite its elevated lipophilicity, clomiphene fulfilled Veber’s criteria without violations, while the reference drugs exceeded the recommended TPSA threshold (>140 Å^2^), suggesting reduced membrane permeability [42,43,44].

These characteristics were reflected in the predicted skin permeation (Table 1). Clomiphene exhibited a log Kp value close to −3, indicating moderate skin permeation compatible with topical formulations, whereas antimoniate and amphotericin B displayed much lower permeability (−11.11 and −11.94, respectively), consistent with their limited penetration across intact skin [45,46].

**Figure 6:**
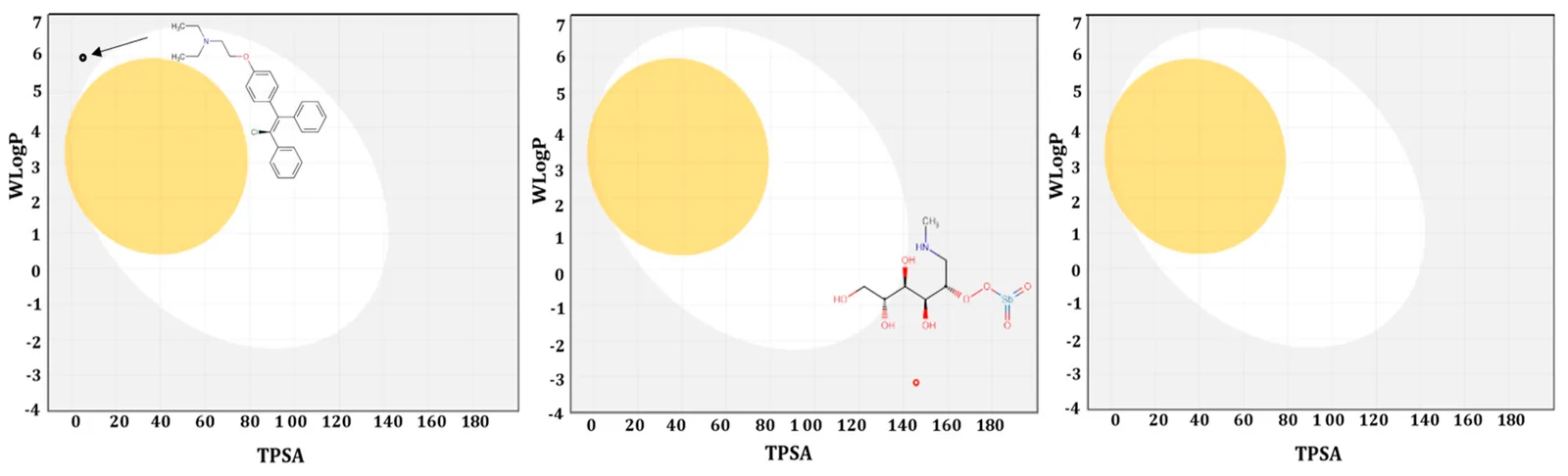
The BOILED-Egg model–WLOGP for the molecules.

The BOILED-Egg model (Figure 6) further supported these findings. Clomiphene was positioned close to the white region, indicating favorable passive absorption and tissue diffusion, whereas antimoniate remained in the gray region, reflecting its high polarity and poor gastrointestinal absorption. Amphotericin B fell outside the model applicability domain because of its large molecular size and amphiphilic character, which is consistent with its clinical intravenous administration [47,48,49].

Predictions obtained with admetSAR 3.0 also supported the favorable pharmacokinetic profile of clomiphene. The compound complied with Lipinski’s rule of five and presented low TPSA (12.47 Å^2^), high lipophilicity (logP = 6.56), satisfactory Caco-2 permeability, and a high probability of oral bioavailability, suggesting efficient passive membrane diffusion. In addition, the absence of P-glycoprotein inhibition may reduce intestinal efflux, whereas the predicted interaction with CYP3A4 indicates metabolism compatible with lipophilic drug candidates [33].

Toxicological predictions indicated no mitochondrial or hemolytic toxicity, low ocular irritation potential, and an estimated oral LD50 of approximately 573 mg/kg, corresponding to moderate acute toxicity. Although its poor aqueous solubility may require formulation strategies, such as lipid-based or nanoencapsulated systems, the combined physicochemical, pharmacokinetic, and toxicity predictions [50,51,52,53,54,55,56,57], support clomiphene as the most promising candidate for topical drug repurposing among the evaluated compounds.

### 3.2. Bioactivity and Molecular Mechanisms

The interaction of candidate compounds with cytochrome P450 (CYP450) isoforms is an important predictor of metabolic stability and potential drug–drug interactions during drug development [58,59,60,61,62]. As summarized in Table 2, clomiphene was predicted to inhibit CYP2C9 while acting as a non-inhibitor/substrate for CYP2D6 and CYP3A4. In contrast, amphotericin B. showed broader CYP inhibition, whereas meglumine antimoniate exhibited fewer predicted metabolic interactions. These results suggest that clomiphene maintains a metabolic profile compatible with drug repurposing, particularly considering its intended topical administration.

Comparative pharmacokinetic predictions further support this strategy. Unlike meglumine antimoniate, which presents negligible skin permeation and complete plasma protein binding, clomiphene combines high lipophilicity with physicochemical properties favorable for local retention in cutaneous tissue. Although standard prediction models indicate limited permeation across intact skin, the disruption of the epidermal barrier in cutaneous leishmaniasis lesions is expected to facilitate drug penetration into infected tissue [63,64,65].

PASS Online predictions (Table 3) reinforced the pharmacological potential of clomiphene. The compound showed high probabilities of interaction with cutaneous CYP isoforms (CYP1A, Pa = 0.928; CYP3A4, Pa = 0.838) together with membrane integrity antagonist activity (Pa = 0.684), supporting its potential for topical application. Amphotericin B. exhibited strong predicted antiprotozoal activity but was also associated with a higher probability of membrane toxicity, whereas meglumine antimoniate was primarily associated with immunomodulatory effects [48,66].

From a pharmacodynamic perspective, clomiphene retained its characteristic activity as a selective estrogen receptor modulator (SERM) while also presenting predicted anti-inflammatory and cytoprotective activities (Table 3). These properties are consistent with previous reports describing the ability of SERMs to modulate inflammatory signaling, tissue remodeling, and membrane-associated pathways, supporting the rationale for repositioning clomiphene as a topical therapeutic candidate for cutaneous leishmaniasis and candidiasis [67,68,69].

### 3.3. Toxicity Effects

The toxicological predictions obtained using GUSAR, ProTox-3, and PreADMET consistently indicated a favorable safety profile for clomiphene compared with meglumine antimoniate and amphotericin B. According to GUSAR (Table 4), clomiphene was classified as OECD Class 5 for oral and subcutaneous administration, indicating low acute toxicity, whereas amphotericin B exhibited higher toxicity, particularly after oral administration (Class 4). These predictions agree with previous experimental reports describing the relatively low systemic toxicity of clomiphene and support its suitability for topical drug repurposing [68,70,71,72,73,74].

**Figure 7:**
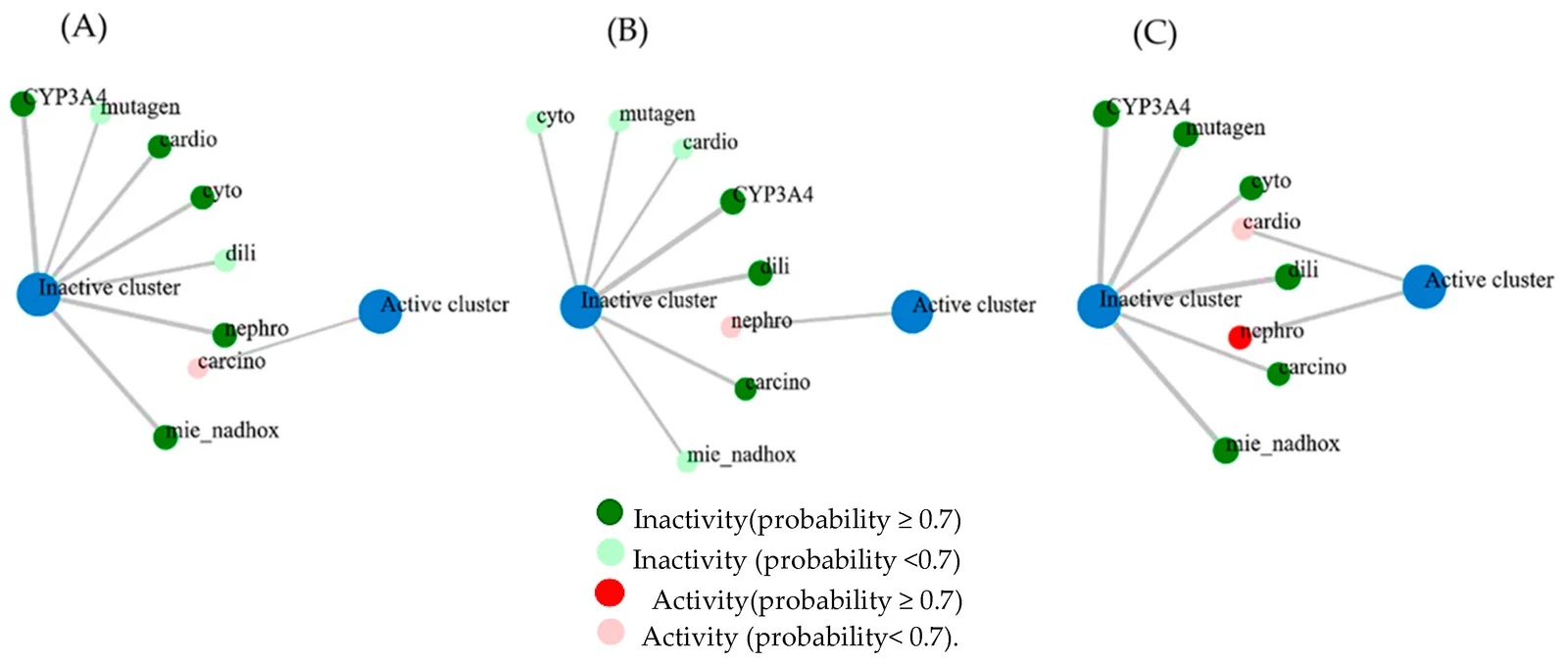
Compound–target map and toxicity prediction. (**A**) Clomiphene, (**B**) Antimoniate, (**C**) Amphotericin B.

ProTox-3 predictions (Figure 7 and Table 5) further demonstrated that clomiphene presented predominantly inactive toxicity clusters, indicating a reduced probability of adverse effects involving major target organs. Although a moderate probability of hepatotoxicity was predicted (22%), the compound showed lower probabilities of nephrotoxicity and cardiotoxicity than the reference drugs.

In contrast, amphotericin B displayed a less favorable profile, with higher probabilities of nephrotoxicity, cardiotoxicity, and oxidative stress-related effects, consistent with its well-established clinical toxicity [75,76,77,78,79,80].

PreADMET predictions corroborated these findings by indicating superior permeability and a lower toxicological risk for clomiphene. The compound exhibited low predicted hERG inhibition risk and negative carcinogenicity in rats (Table 6), whereas amphotericin B showed a positive carcinogenicity prediction and a less favorable toxicity profile. Although clomiphene was predicted to interact with CYP2C9 and CYP3A4, its intended topical administration is expected to minimize the clinical relevance of systemic drug–drug interactions.

Overall, the convergence of three independent computational platforms supports clomiphene as the compound with the most balanced safety profile among the evaluated drugs, reinforcing its potential for topical repurposing in the treatment of cutaneous leishmaniasis and candidiasis.

### 3.4. Molecular Interaction

**Figure 8:**
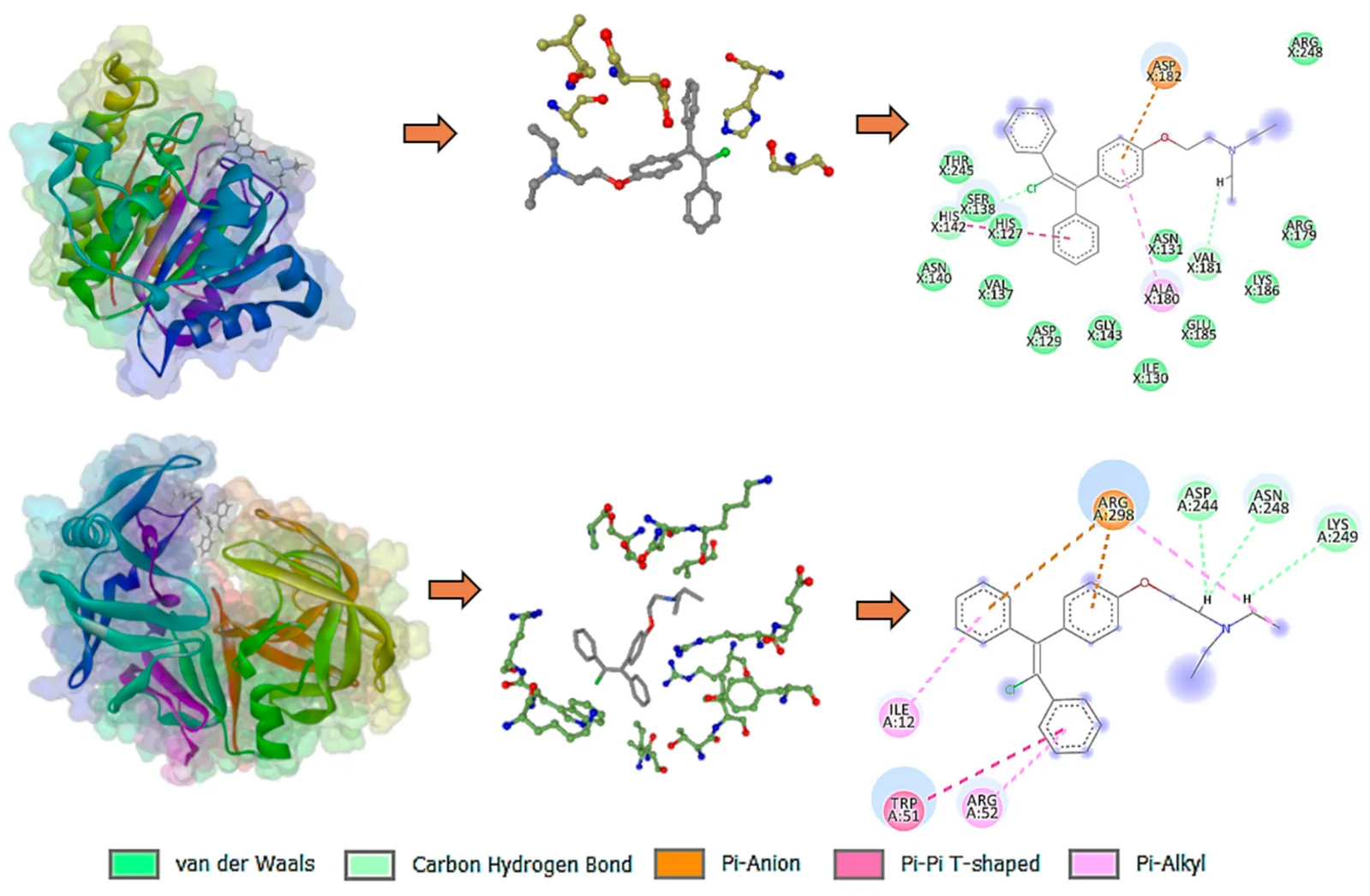
Protein–ligand complexes of clomiphene–arginase (PDB: 5HJA) and clomiphene–Sap5 (PDB: 2QZX): main interactions within the active site.

Figure 8 shows the binding modes of clomiphene within the catalytic sites of *Leishmania* sp. arginase (5HJA) and Sap5 (Secreted Aspartic Proteinase 5) from *Candida albicans*. Molecular docking analysis revealed that clomiphene exhibits a favorable predicted binding affinity toward Sap5, with a binding energy of −8.96 kcal/mol, indicating a spontaneous interaction and supporting its potential for further experimental validation.

The predicted binding pose positions the ligand within the Sap5 catalytic pocket, where it establishes a complementary network of interactions, including hydrogen bonds with polar residues, π–cation electrostatic contacts, and a T-shaped π–π interaction. The diversity of these specific interactions suggests effective molecular recognition and contributes to the binding specificity of the ligand within the active site [81,82].

The observed interaction distances (2.5–3.5 Å, Table 7) fall within the typical range for hydrogen bonds, indicating efficient polar anchoring and proper ligand orientation, which are expected to enhance binding specificity [83,84]. Hydrogen bonds involving Asp244, Asn248, and Lys249 represent important stabilization points, whereas the dual π–cation interaction with Arg298 indicates a significant electrostatic contribution to binding. In addition, the T-shaped π–π interaction with Trp51 further stabilizes ligand accommodation within the hydrophobic region of the active site, resulting in a favorable balance between polar and hydrophobic interactions that supports complex stability [85].

This interaction pattern suggests that clomiphene exhibits favorable structural complementarity with the target protein, supported by polar and aromatic interactions that may contribute to binding affinity and specificity (Table 8). These findings reinforce the potential of clomiphene for antifungal drug repurposing, particularly in light of its established role as a selective estrogen receptor modulator (SERM) and its previously reported activity against fungal biofilms and enzymes. Nevertheless, molecular docking provides only preliminary evidence of ligand–target interactions, and these findings should be validated through molecular dynamics simulations, enzymatic assays, and *in vitro* studies to confirm the compound’s activity and selectivity [86,87,88].

The clomiphene–5HJA (Leishmania) and clomiphene–Sap5 (2QZX, *C. albicans*) complexes exhibited strong predicted binding affinities (ΔG = −9.21 and −8.96 kcal/mol, respectively), with values well below the commonly accepted threshold for promising compounds (≤−7.0 kcal/mol). In contrast, docking scores for less active compounds generally range from −3.80 to −6.90 kcal/mol [89,90]. 

The interaction with Sap5 is particularly noteworthy because of its pronounced van der Waals contribution, reflecting efficient occupation of the hydrophobic binding pocket. This interaction is further stabilized by T-shaped π–π stacking with Trp51 and additional nonpolar contacts, as summarized in Table 7. Energy decomposition analysis indicates that hydrophobic interactions are the primary contributors to complex stability, consistent with previous studies [88]. Furthermore, the favorable electrostatic component supports complex stabilization through multiple hydrogen bonds involving Asp244, Asn248, and Lys249, together with π–cation interactions with Arg298.

For the 5HJA (Leishmania) complex, favorable hydrophobic and electrostatic contributions also support the high binding affinity, demonstrating the ability of clomiphene to establish stable interactions with both protozoan and fungal targets.

Overall, the docking scores of −9.21 and −8.96 kcal·mol^−1^ for 5HJA and Sap5, respectively, further support the biotechnological potential of clomiphene and justify its continued investigation as a promising candidate for the development of new therapeutic strategies against leishmaniasis and candidiasis caused by *Candida albicans*.

### 3.5. Spectroscopic Analysis by ATR-FTIR

**Figure 9:**
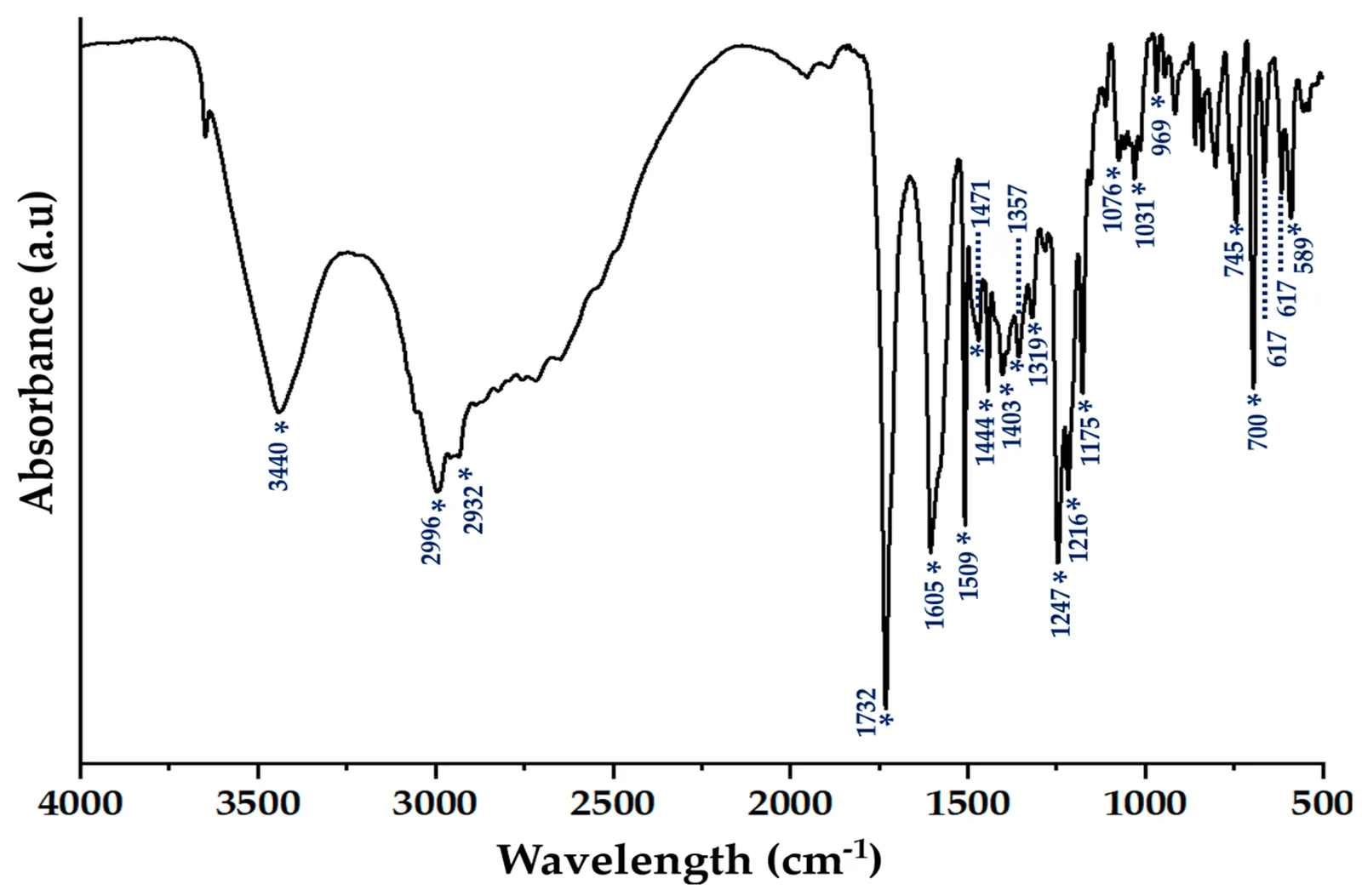
Fourier-transform infrared (FTIR) spectrum of clomiphene citrate, highlighting the main absorption bands. (*) indicate the wavenumber positions of the main absorption bands.

FTIR analysis confirmed the presence of functional groups characteristic of the clomiphene citrate structure, through the absorption bands observed in the spectrum (Figure 9). The band at 3440 cm^−1^ was attributed to O–H stretching, indicating a broad and moderately intense absorption related to the citric acid present in the clomiphene citrate form and/or residual moisture in the sample. This behavior is compatible with the presence of hydroxyl groups and hydrogen bonds, which tend to broaden this vibration in the 3200 to 3500 cm^−1^ region. The bands at 2996 and 2932 cm^−1^ correspond to C–H stretchings, associated with aliphatic and/or aromatic groups, which are characteristic of carbon chains in different structural environments.

The absorption at 1732 cm^−1^ was related to C=O stretching, compatible with the carbonyl group of the citrate and the formation of the clomiphene citrate salt. This band is relevant because it highlights the carbonyl group of citric acid, whose vibration is typically intense within this range. The bands at 1605 and 1509 cm^−1^ indicate C=C stretchings of the aromatic rings, reinforcing the contribution of the phenyl moieties present in the clomiphene structure. Furthermore, the bands at 1471, 1444, and 1403 cm^−1^ are associated with C–H deformations and contributions from CH_2_/CH_3_ groups, as well as aromatic ring vibrations. Similarly, the bands at 1357 and 1319 cm^−1^ can be attributed to C–H deformations and vibrational modes of the citrate, including contributions from the molecular backbone and potential δ(C–O–H)-type vibrations. In the 1247 and 1216 cm^−1^ range, C–O/C–O–C vibrations and possible C–N contributions are observed, which are characteristic of the fingerprint region. The band at 1216 cm^−1^ is especially compatible with C–O–C stretching, whereas the band at 1175 cm^−1^ reinforces the presence of bonds involving oxygen and nitrogen. The bands at 1076 and 1031 cm^−1^ can also be attributed to C–O, C–C, and C–N vibrations, which are typical of this same spectral region.

Finally, the bands at 969 and 745 cm^−1^ reflect out-of-plane aromatic C–H deformations and potential C–Cl contributions, which are consistent with substituted benzene rings in the clomiphene citrate structure. The band at 700 cm^−1^ corresponds to the out-of-plane C–H deformation of a mono- or poly-substituted benzene ring, whereas the bands at 666, 617, and 589 cm^−1^ indicate C–Cl stretching modes and skeletal deformations of substituted aromatic rings.

### 3.6. In Vitro Study on Leishmania Promastigote Forms

To assess the antileishmanial potential of clomiphene citrate, its activity was investigated on promastigote forms, an initial screening step that guides subsequent studies against the parasite’s intracellular form.

**Figure 10:**
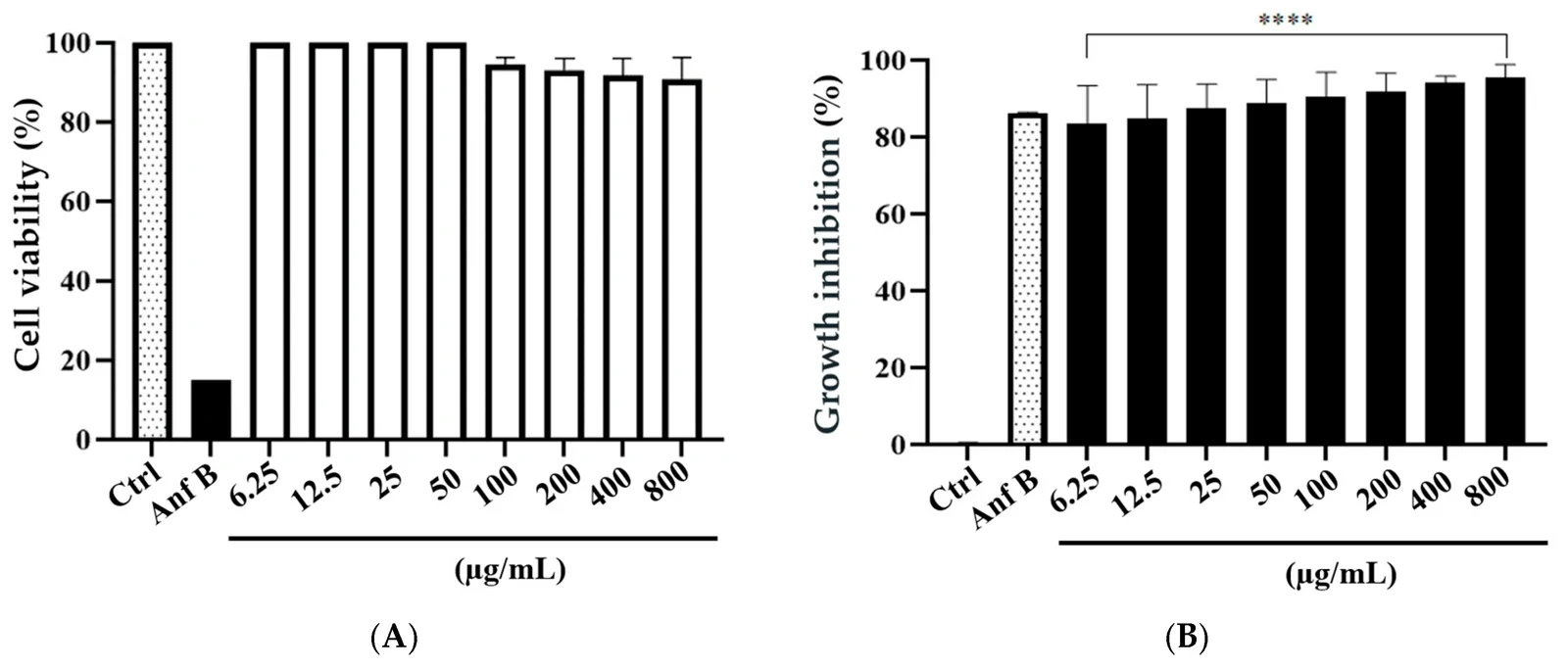
*In vitro* evaluation of the antileishmanial activity and cytotoxicity of clomiphene citrate against *Leishmania amazonensis*. (**A**) Cell viability (%) after treatment with clomifene citrate at 6.25, 12.5, 25, 50, 100, 200, 400, and 800 µg/mL, compared with control (Ctrl) and amphotericin B (AmB). (**B**) Growth inhibition (%) of Leishmania after treatment with the same concentrations, compared with control (Ctrl) and amphotericin B (AmB). Legend: **** (*p* < 0.00001).

The cytotoxicity of clomiphene citrate toward macrophages suggests a favorable biological profile for preclinical advancement. In the MTT assay with murine macrophages (Figure 10A), clomiphene citrate maintained cell viability above 80% even at a concentration of 800 μg/mL, which is close to the negative control, indicating low host toxicity and a CC_50_ > 490 μg/mL (Table 9). In contrast, amphotericin B reduced viability to less than 20%, confirming its expected cytotoxicity in macrophages. This profile, associated with an IC_50_ = 0.27 ± 0.032 μg/mL against promastigotes, results in a selectivity index > 50, which is above the threshold considered promising for preclinical screening. Previous studies reinforce this selectivity by showing preservation of macrophage morphology up to 6 μM of clomiphene and action against intracellular amastigotes of *L. mexicana* (IC_50_ = 2.8 μg/mL) [14].

In the growth inhibition of L. amazonensis promastigotes (Figure 10B), clomiphene citrate exhibited a greater inhibitory effect than Amphotericin B., even at the lowest concentration tested (6.25 μg/mL), displaying a dose-dependent response up to 800 μg/mL. Its potency (IC_50_ = 0.27 ± 0.032 μg/mL) was superior to that of amphotericin B (IC_50_ = 0.62 ± 0.14 μg/mL), indicating better performance of clomiphene to inhibit growth after 48 h of incubation. 

In turn, the SI > 50 aligns with the WHO guidelines for preclinical candidates (SI > 10 is considered viable; >100 is ideal), thereby reducing toxicological risks and potentially justifying complementary tests such as hemocompatibility, HET-CAM, PK/PD in vivo. For instance, recent literature [91] exemplifies a similar selectivity in an anti-*L. amazonensis* flavonoid fraction (CC_50_ of 251 μg/mL in RAW 264.7 macrophages, SI = 12), which suggests combinations with antimonials to optimize therapies. Thus, these data position clomiphene citrate (CC_50_ > 490 μg/mL, SI > 50) as an innovative candidate for *in vivo* validation in the present study. The high CC_50_ positions clomiphene as a less nephrotoxic alternative for vulnerable populations. In this way, clomiphene citrate stands out for its greater therapeutic margin, favoring its repositioning given the clinical safety already established in infertility treatments with doses of 25–50 mg/day [92]. Studies such as that by author et al. [93] describe the mechanism for SERMs as involving mitochondrial and parasitic sphingolipid disruption without significant macrophage necrosis. This graphical dynamic reinforces the therapeutic potential of clomiphene, achieving an inhibitory effect equivalent or superior to that of Amphotericin B. across all dilutions, with a clear advantage at low concentrations, which is ideal for potential drug repurposing.

### 3.7. In Vitro Study for Biofilm Inhibition

**Figure 11:**
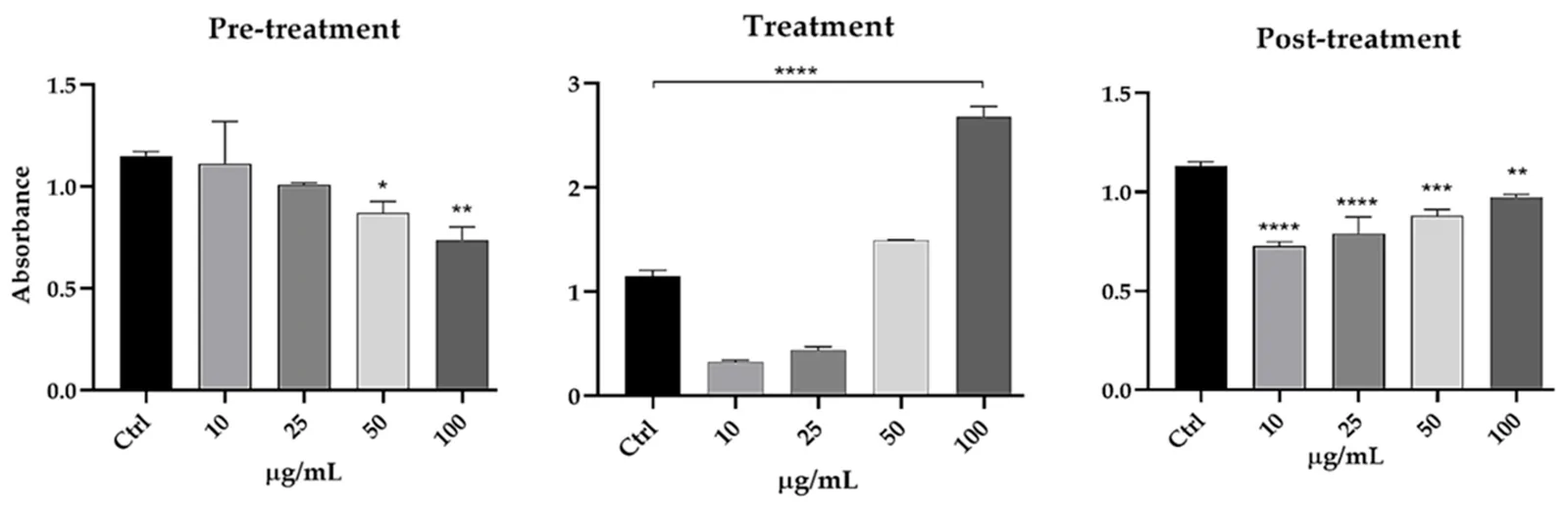
Post-treatment absorbance of control and treated groups with 10, 25, 50, and 100 μg/mL. Data are expressed as mean ± standard deviation. Statistical comparisons were performed using one-way ANOVA followed by Dunnett’s test, taking the control group as a reference. * *p* < 0.05, ** *p* < 0.01, *** *p* < 0.001, and **** *p* < 0.0001 compared to the control.

As observed in Table 10 and corroborated by the graphical profile in Figure 11, clomiphene citrate alone exhibited a paradoxical behavior in the treatment (B) and post-treatment (C) groups, while low doses were effective: 50 μL and 100 μL induced a biomass increase of 29.94% and 133.22%, respectively.

In the pre-treatment, the results suggest that clomiphene citrate interferes more efficiently with the initial stages of biofilm formation, when the yeasts are still in the contact phase with the drug prior to stable adhesion to the surface. This is consistent with the literature describing C. albicans biofilm formation as a sequential process, initiated by initial adhesion and followed by proliferation, maturation, and dispersion, with the adhesion phase being particularly sensitive to external interventions [94].

Adhesion and maintenance proteins play distinct roles during the first hours of biofilm formation, reinforcing the possibility of greater susceptibility before the biofilm consolidates [95]. In this context, initial adhesion is considered a critical stage for therapeutic resistance; in C. albicans, for instance, the proteins Bcr1, Als1, Als3, and Hwp1 participate in cell attachment and retention [96]. In early biofilms, the extracellular matrix is still poorly developed, which reduces the drug’s action barrier. In contrast, mature biofilms possess the ability to sequester drugs, thereby limiting their penetration, although this effect is less pronounced during the initial adhesion phase [97]. Thus, when the compound is applied prior to substrate attachment, it can compromise the formation of the biofilm base, explaining the dose-dependent inhibition that becomes more evident at high concentrations.

A reduction in biofilm is observed at low concentrations of clomiphene citrate, whereas an increase occurs at high doses, highlighting a non-linear response. This behavior may be related to stress response pathways regulated by Hsp90 and calcineurin, which promote adaptation, survival, and antifungal tolerance in biofilms [98,99]. Thus, the increase at high doses may reflect an adaptive response.

Exposure to high concentrations of the substance may also induce defense mechanisms linked to the cell wall and the recruitment of persister cells, which survive high antifungal concentrations [100]. Nevertheless, the activity at low concentrations remains relevant as it does not depend on direct toxicity [101,102], reinforcing its potential as a practical solution. In the post-treatment, a less pronounced reduction was observed, possibly due to the already consolidated matrix [103,104]. In *C. albicans*, this matrix, rich in glucans, mannans, and proteins, hinders drug diffusion and protects internal cells, contributing to high tolerance even in the absence of mandatory genetic resistance [105]. 

Thus, a lower efficacy in the post-treatment reflects the barrier imposed by the matrix and physiological factors, such as lower metabolic activity and the presence of persister cells. Taken together, clomiphene citrate exhibited a greater effect in the treatment group during adhesion, an intermediate effect in the post-treatment group, and a dose-dependent inhibition in the pre-treatment group, demonstrating a relevant therapeutic window at low doses.

## 4. Conclusions

Computational analyses performed using SwissADME and admetSAR 3.0 indicated that clomiphene possesses a favorable pharmacokinetic profile, including high lipophilicity, moderate skin permeability, and predicted good oral bioavailability and intestinal absorption. PASS Online suggested relevant biological activities, whereas toxicity assessments using GUSAR, ProTox-3, and PreADMET predicted a favorable safety profile, characterized by low oral and environmental toxicity, a low probability of cardiotoxicity and nephrotoxicity, and no predicted carcinogenicity. Experimental characterization confirmed the identity of clomiphene by ATR-FTIR spectroscopy, while the biological assays demonstrated antileishmanial and antifungal activities. Molecular docking predicted favorable interactions of clomiphene with Leishmania sp. L-arginase and Candida albicans Sap5, providing structural support for its potential dual mechanism of action. Furthermore, the *in vitro* assays revealed potent leishmanicidal activity (IC_50_ = 0.27 μg/mL), a high selectivity index (SI > 50), and inhibition of the early stages of C. albicans biofilm formation. Taken together, these findings provide preliminary evidence supporting the potential of clomiphene citrate as a candidate for drug repurposing against tegumentary leishmaniasis and candidiasis caused by *Candida albicans.* However, additional mechanistic, pharmacokinetic, and *in vivo* studies are required to further establish its efficacy and safety before clinical application.

## Figures and Tables

**Table 1:** Interpretation scale for log K_p_.

log K_p_ Value (cm/s)	Technical Interpretation	Skin Permeation Capability
>−4	High permeability	Readily crosses the stratum corneum
−6 ≥ −4	Moderate permeability	Ideal profile for most topical and transdermal drugs
−8 ≥ −6	Low permeability	Poorly crosses intact skin.
≤−8	Very low permeability	Extremely poor penetration

**Table 2:** Evaluation of the interaction of new active compounds.

Parameter	Clomiphene	Antimoniate	Amphotericin B
CYP2C19 inhibition	Non-inhibitor	Non-inhibitor	Inhibitor
CYP2C9 inhibition	Inhibitor	Non-inhibitor	Inhibitor
CYP2D6 inhibition	Non-inhibitor/Substrate	Inhibitor/Substrate	Inhibitor
CYP3A4 inhibition	Non-inhibitor/Substrate	Non-inhibitor/Substrate	Inhibitor
Plasma protein binding	92.9%	100.0%	39.0%
Skin permeability (log Kp)	−3.64	−11.34	−11.94
Parameter	Clomiphene	Antimoniate	Amphotericin B.

**Table 3:** Relevant PASS Online data for Clomiphene, Antimoniate, and Amphotericin B. Syst. (systemic), main CYPs (Pa > 0.8).

Activity	Clomiphene (Topical)	Antimoniate (Systemic)	Amphotericin B (Systemic)
Antiparasitic action	Indirect	Direct	Direct (ergosterol binding)
Anti-inflammatory	Yes (Pa: 0.936)	Yes (Pa: 0.936)	Yes (Pa: 0.330)
Anti-inflammatory	Yes (Pa: 0.936)	Yes (Pa: 0.936)	Yes (Pa: 0.330)
Skin permeation	Favorable	Unlikely	No (systemic use)
Cytoprotection	Yes (Pa: 0.586)	Not highlighted	Yes (Pa: 0.939)
Adverse effects	Low (topical)	High (renal)	High (nephrotoxicity)
Main CYPs	CYP1A (0.928), CYP2C12 (0.949), CYP3A4 (0.838)	CYP2C12 (0.792)	CYP3A4 (0.838)
Topical use	Viable	Viable	Experimental

**Table 4:** Acute toxicity in rats (GUSAR). IP: intraperitoneal route of administration; IV: intravenous route of administration; Oral route: oral administration; SC: subcutaneous route of administration; C: model-defined classification level.

Route/Drug	Clomiphene	Amphotericin B	Antimoniate	OECD Class (Clomiphene)
Oral	2013 (Class 5)	1031 (C.4)	2178 (C.5)	Practically non-toxic
IV	30,500 (Class 3)	25,770 (C.3)	831 (C.5)	Moderate
IP	250 (Class 4)	937 (C.5)	405 (C.4)	Slightly toxic
SC	1030 (Class 5)	323 (C.4)	2172 (C.5)	Practically non-toxic

**Table 5:** Representation of the predicted toxicity probability by ProTox-3.

Category/Target		Clomiphene	Antimonate	AmB
Organ toxity	Hepatotoxicity	22%	8%	3%
	Nephrotoxicity	57%	64%	77%
	Cardiotoxicity	33%	41%	34%
Toxicity pathways	Cytotoxicity	21%	32%	17%
	Carcinogenicity	38%	28%	27%
	Mutagenicity	30%	32%	11%
Molecular activity	CYP3A4	3%	1%	2%
	Enzyme (NADHOX)	15%	37%	44%

**Table 6:** Toxicological parameters predicted by PreADMET for clomiphene, antimonite and amphotericin B.

Toxicity	Clomiphene	Antimoniate	Amphotericin B
Carcino_Rat	Negative	Out of range	Positive
hERG inhibition	Low risk	Medium risk	Ambiguous
Algae toxicity	1.60 × 10^−5^	0.00501397	0.000376181
Daphnia toxicity	0.0084237	23.5578	0.365508
Medaka toxicity	0.000220139	623.482	0.418441
Minnow toxicity	3.55 × 10^−29^	207.773	1.82514

**Table 7:** Molecular interactions identified in the docking of clomiphene with Candida albicans aspartic proteinase Sap5 (PDB ID: 2QZX).

Ligand	Energy Score	Residues	Nature	Molecular Interaction	Interaction Distance (Å)
Ligand_1 (5HJA)	−9.211	HIS142	Hydrogen Bond	Carbon Hydrogen Bond	3.54415
		VAL181	Hydrogen Bond	Carbon Hydrogen Bond	3.0208
		ASP182	Electrostatic	Pi-Anion	4.97025
		ALA180	Hydrophobic	Pi-Alkyl	5.35617
Ligand_1 (SAP5)	−8.956	ASP244	Hydrogen Bond	Carbon Hydrogen Bond	2.5581
		ASN248	Hydrogen Bond	Carbon Hydrogen Bond	3.02159
		LYS249	Hydrogen Bond	Carbon Hydrogen Bond	2.84928
		ARG298	Electrostatic	Pi-Cation	3.60564
		ARG298	Electrostatic	Pi-Cation	4.51217
		ARG298	Electrostatic	Pi-Cation	4.88847
		TRP51	Hydrophobic	Pi-Pi T-shaped	5.63721

**Table 8:** Molecular docking thermodynamic parameters of clomiphene with 5HJA (Leishmania) and Sap5 (2QZX, *Candida albicans*).

Thermodynamics Parameter	Values (kcal·mol^−1^)	
	5HJA	SAP5
Binding Score (ΔG)	−9.211	−8.956
Interaction Energy	−26.805	−25.459
Van der Waals Energy	−23.823	−21.138
Electrostatic Energy	−2.982	−4.321

**Table 9:** Median inhibitory and cytotoxic concentrations, and selectivity index of clomiphene citrate and amphotericin B against *L. amazonensis.*

CI_50_	CC_50_	SI	CI_50_ AmB B
0.27 ± 0.032	490 ± 0.024	>50	0.62 ± 0.14

**Table 10:** Percentage of reduction/increase in biofilm during testing of substances to verify Candida albicans biofilm inhibition. * biofilm increase relative to the control group.

Treatment	Test	10 μL	25 μL	50 μL	100 μL
Pre-treatment	Clomiphene	3.40%	12.37%	24.24%	35.92%
Treatment	Clomiphene	71.81%	61.93%	29.94% *	133.22% *
Post-treatment	Clomiphene	36.68%	29.95%	21.93%	13.67%

## Data Availability

The raw data supporting the conclusions of this article will be made available by the authors upon request.

## Supplementary Materials

The following supporting information can be downloaded at: https://www.mdpi.com/article/10.3390/biom16081133/s1, Figure S1. Validation of the docking protocol by redocking the co-crystallized ligands into the binding sites of (A) Sap5 (PDB ID: 2QZX) and (B) Leishmania L-Arginase (PDB ID: 5HJA). Superposition of the crystallographic and redocked ligand conformations is shown for each target. In (A), the crystallographic ligand is displayed in blue, whereas the redocked pose is shown in orange. In (B), the crystallographic ligand is displayed in yellow, whereas the redocked pose is shown in purple. The redocking procedure yielded RMSD values of 1.4145 Å for Sap5 and 1.6166 Å for L-Arginase. Figure S2. Grid box used for redocking validation in (A) Sap5 (PDB ID: 2QZX) and (B) Leishmania L-Arginase (PDB ID: 5HJA).
