# Supplementary material for: Repurposing of Clomiphene Citrate and Its Antileishmanial and Antifungal Activities: In Silico and In Vitro Studies
Source: Biomolecules. 2026 Aug 3;16(8):1133. doi: 10.3390/biom16081133 (PMC13511042; doi:10.3390/biom16081133)
Supplement: Supplementary file 1 [file biomolecules-16-01133-s001.zip › biomolecules-4452446-supplementary.pdf]

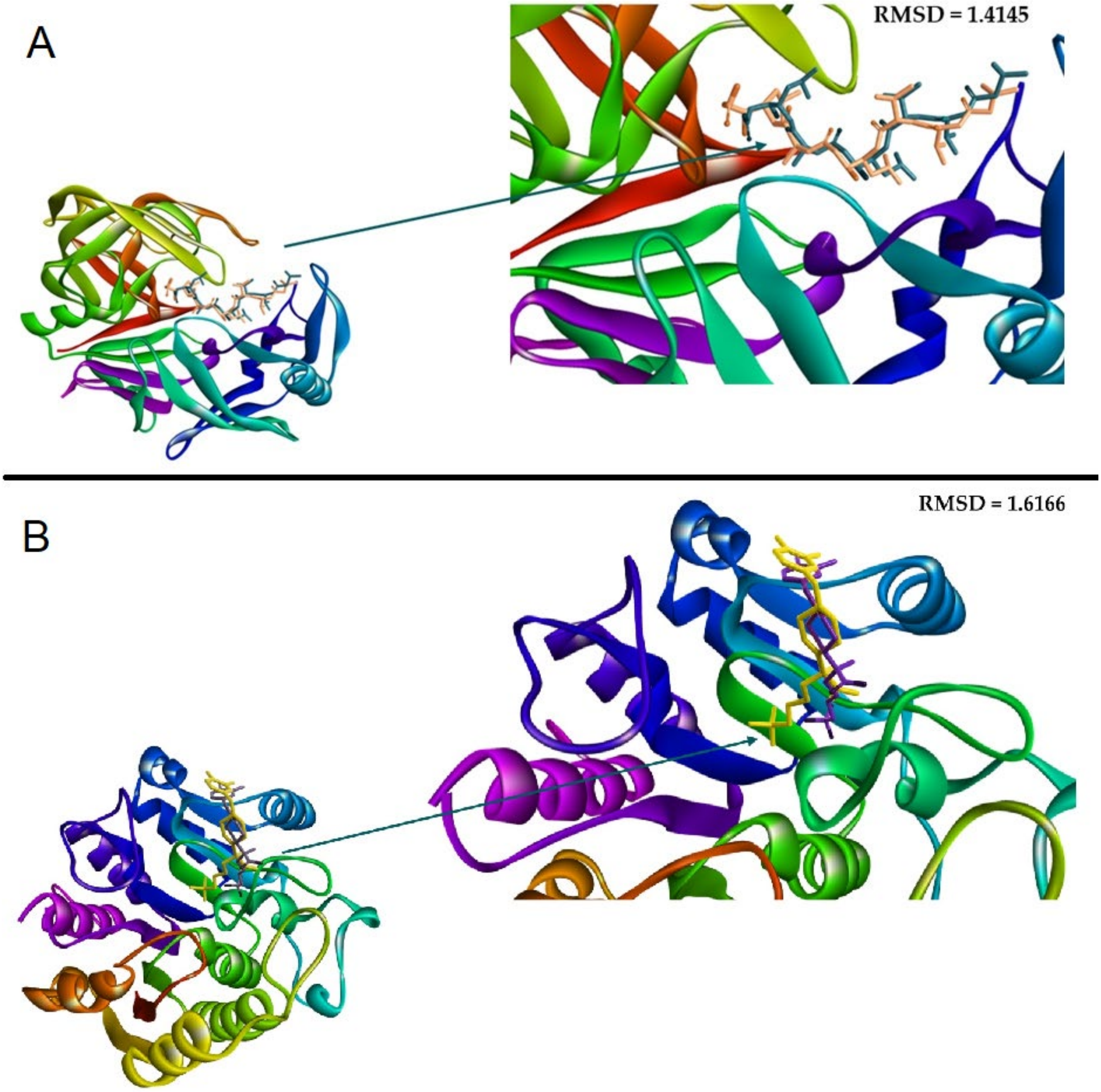

Figure S1. Validation of the docking protocol by redocking the co-crystallized ligands into the binding sites of (A) Sap5 (PDB ID: 2QZX) and (B) Leishmania L-Arginase (PDB ID: 5HJA). Superposition of the crystallographic and redocked ligand conformations is shown for each target. In (A), the crystallographic ligand is displayed in blue, whereas the redocked pose is shown in orange. In (B), the crystallographic ligand is displayed in yellow, whereas the redocked pose is shown in purple. The redocking procedure yielded RMSD values of 1.4145 Å for Sap5 and 1.6166 Å for L-Arginase.

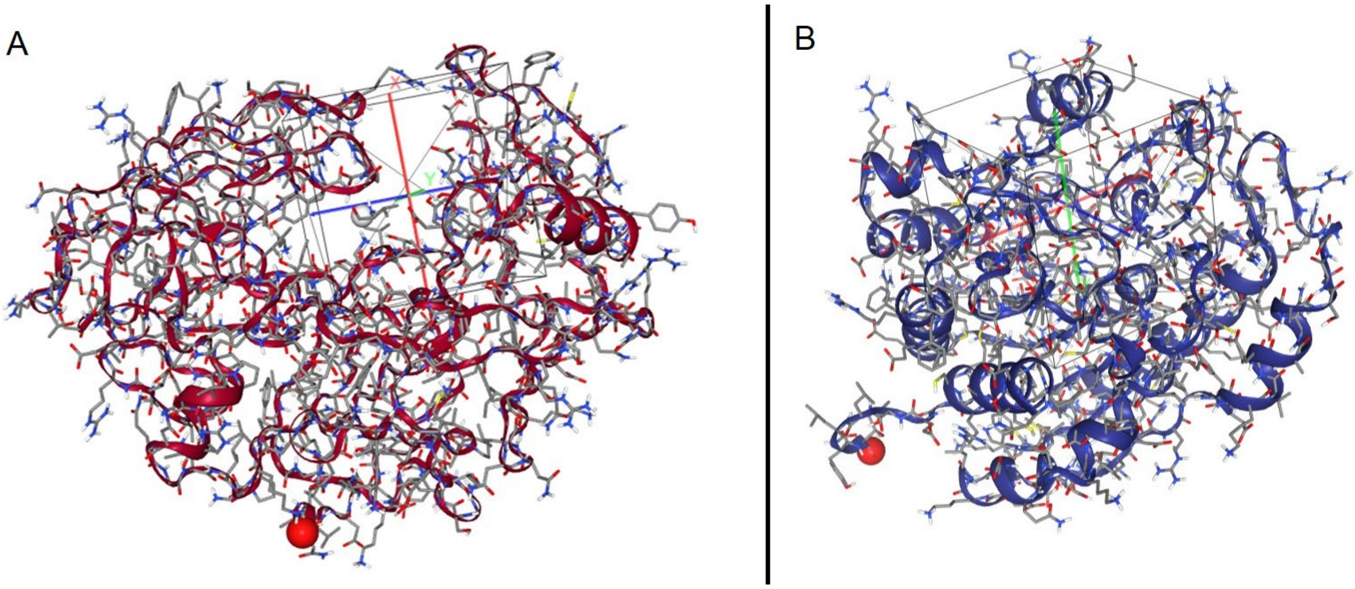

Figure S2. Grid box used for redocking validation in (A) Sap5 (PDB ID: 2QZX) and (B) Leishmania L-Arginase (PDB ID: 5HJA).
